# Actin Takes the Steering Wheel: A New Mitotic Safeguard in the Early Embryo

**DOI:** 10.34133/research.1050

**Published:** 2025-12-22

**Authors:** Yingqi Zhang, Shukai Zhu, Fangwei Wang

**Affiliations:** ^1^ Department of Gynecologic Oncology, Women’s Hospital, School of Medicine and MOE Laboratory of Biosystems Homeostasis & Protection, Life Sciences Institute, Zhejiang University, Hangzhou 310058, China.; ^2^ Zhejiang Key Laboratory of Geriatrics, Affiliated Zhejiang Hospital, School of Medicine, Zhejiang University, Hangzhou 310058, China.; ^3^ State Key Laboratory of Transvascular Implantation Devices, Hangzhou 310009, China.; ^4^ Cancer Center, Zhejiang University, Hangzhou 310058, China.

## Abstract

Early mammalian embryos undergo crucial cell divisions despite lacking fully functional centrosomes, presenting a paradox of how mitotic fidelity is maintained. This commentary discusses a seminal study by Hernandez et al. that overturns the microtubule-centric model of early embryonic mitosis by revealing 2 essential, complementary F-actin networks. First, a formin-dependent nuclear F-actin network, cross-linked by anillin, positions prophase chromosomes and then drives their centripetal congregation upon nuclear envelope breakdown via a novel depolymerization-based contraction mechanism. Second, a branched, Arp2/3-dependent peri-spindle actin shell acts as a physical brake to restrain spindle elongation. Using advanced live imaging and targeted perturbations in mouse and human embryos, the study demonstrates that these actin assemblies collectively ensure accurate chromosome segregation by compensating for weak centrosomal activity. These findings redefine the mechanical landscape of early embryonic cell division, with profound implications for understanding the origins of aneuploidy and advancing reproductive medicine.

In early embryonic development, microfilaments (F-actin) and microtubules constitute the core cytoskeletal elements with distinct yet complementary functions. F-actin typically mediates cortical integrity, cytokinesis, and cellular morphogenesis, while microtubules form the mitotic spindle and facilitate intracellular transport [1,2].

Early embryonic cell divisions are high-stakes events. A single mis-segregated chromosome in the zygote or early cleavage stages can produce aneuploidy and derail development [3], yet the first mammalian divisions occur in an unusual cellular context: mouse embryos lack fully functional centrosomes until the blastocyst due to their elimination during oogenesis [4], while human zygotes, despite inheriting sperm-derived centrioles, frequently display multipolar spindles, suggesting compromised centrosomal activity [5]. This specialized adaption may optimize rapid embryonic cell cycles. How, then, do these large, acentrosomal cells reliably organize chromosomes and build spindles? Hernandez et al. [6] revisit that paradox and reveal a striking, actin-based solution: 2 distinct F-actin assemblies, a contractile nuclear actin network and a branched peri-spindle actin shell, work together to position chromosomes and to restrain spindle growth, ensuring mitotic fidelity in preimplantation embryos (Fig. 1). This research offers a novel perspective with profound implications for reproductive medicine and developmental biology.

**Fig. 1.**
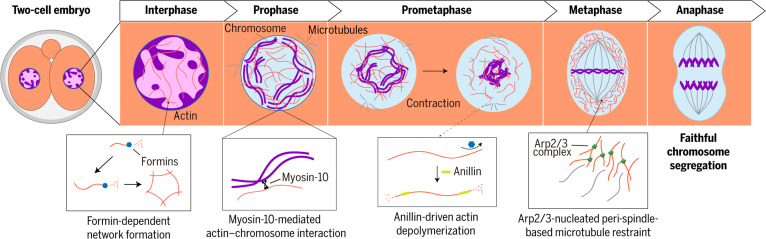
Actin networks safeguard mitotic fidelity in early embryos. This model illustrates the 2 actin-based mechanisms identified by Hernandez et al. that compensate for absent or compromised centrosomes during preimplantation development. A formin-dependent nuclear F-actin network persists from interphase into mitosis, where it positions prophase chromosomes at the nuclear periphery through myosin-10. Following nuclear envelope breakdown (NEBD), anillin-mediated filament disassembly drives network contraction, aiding chromosome organization. In metaphase, branched Arp2/3-generated peri-spindle actin restrains microtubule overgrowth. Together, these actin assemblies ensure accurate chromosome segregation despite the lack of canonical centrosomal machinery.

Classical models place microtubules and centrosomes at the center of mitotic organization: centrosome-nucleated microtubules rapidly search and capture kinetochores after nuclear envelope breakdown (NEBD), while cortical pulling forces mediated by dynein at the cell cortex orient the spindle [7,8]. Cortical F-actin interacts with astral microtubules to generate these positioning forces, and a ringlike F-actin structure in metaphase HeLa cells further contributes to spindle positioning [9,10]. However, in early mammalian embryos, canonical centrosomal function is absent or compromised. Prior work had hinted that actin might play roles in oocyte meiosis and in some nonmammalian mitoses [10–12], yet the idea that actin could substitute for centrosomal functions during the earliest embryonic mitoses remained controversial. Sparse, hard-to-see actin filaments and the dominance of the microtubule narrative left a mechanistic blind spot: what, if anything, organizes chromosomes at and immediately after NEBD in these acentrosomal divisions, and how is mitotic fidelity maintained? The paper directly addresses that unresolved question.

The authors employed high-resolution live imaging, targeted protein perturbations, and quantitative image analysis to visualize and dissect actin’s contributions through mitosis in mouse and (where possible) human preimplantation embryos. The experimental design included fluorescent labeling of F-actin with UtrCH-GFP and phalloidin, and chromosomes with H2B-mRFP1 or SPY650-DNA, and dynamic tracking with sub-5-s temporal resolution. They used targeted degradation (TRIM-Away) and dominant-negative constructs (myosin-10 ΔHL), as well as nuclear-targeted actin mutants (NLS-actin G13R and S14C) to manipulate nuclear filaments specifically. Complementary pharmacology (cytochalasin D, jasplakinolide, latrunculin A, SMIFH2, and CK-666) and expression of Arp2/3 modulators allowed separate interrogation of filament turnover, formin-dependent nucleation, branched-filament assembly, and motor activity. Finally, quantitative metrics—convex-hull scattering volume, particle tracking of actin network nodes, and peri-spindle intensity profiles—provided rigorous, time-resolved readouts of chromosome arrangement and spindle geometry. This approach enabled visualization of previously unobserved actin structures and testing of causal relationships.

One core finding of this study is that a nuclear F-actin network organizes chromosomes before the spindle assembly. This builds on previous observations of actin-mediated chromosome organization in oocytes [11,13]. During interphase and prophase, the authors observed a dense, cable-like F-actin network that traverses the nucleus and increases in density in prophase. Prophase chromosomes sit at the nuclear periphery, tethered to this network via the unconventional motor myosin-10. After NEBD, the network undergoes a rapid, centripetal contraction that gathers chromosomes toward the cell center, a process that completes within minutes and precedes robust spindle assembly. Crucially, this contraction is microtubule independent and does not require nonmuscle myosin II; instead, contraction is driven by filament disassembly (net depolymerization) and requires the diffusible cross-linker anillin. The authors addressed potential drug nonspecificity by using multiple agents with different mechanisms: both cytochalasin D (prevents polymerization) and jasplakinolide (prevents depolymerization) impaired contraction, while latrunculin A (enhances depolymerization) accelerated it. Perturbations that eliminate or hyperstabilize the nuclear filaments (cytochalasin D, NLS-actin G13R or S14C, and jasplakinolide) or that deplete anillin impair centripetal movement, delay congression, prolong mitosis, and increase chromosome segregation errors. These data show that a depolymerization-driven, anillin-cross-linked nuclear F-actin network positions chromosomes and reduces the burden on a slow, acentrosomal spindle.

Another core finding of this study is that a peri-spindle, branched F-actin shell limits spindle elongation. After spindle assembly, the authors identified a second actin structure: a dense, patch-like peripheral F-actin surrounding the metaphase spindle. Similar structures have been observed in mouse oocytes and *Xenopus* systems [12,14]. This peri-spindle network is enriched for Arp2/3 (Arp3) and appears to be nucleated locally by RanGTP signaling from chromatin. Disrupting Arp2/3 (CK-666) or depleting peri-spindle actin (memb-VCA sequestration) produces oversized, elongated spindles and fragmented poles, phenotypes consistent with a loss of a physical brake that normally restrains microtubule overgrowth. Thus, branched actin acts as a provisional, physical barrier that modulates spindle dimensions in the absence of canonical centrosomal length-control mechanisms. An important question is whether anillin, which cross-links the nuclear network, also coordinates with cortical F-actin structures. While not addressed in this study, anillin’s known role in cytokinesis and cortical organization suggests that it could potentially integrate multiple actin networks.

Two mechanistic novelties of these findings stand out. First, contractility driven by filament disassembly, rather than myosin-driven sliding, powers the nuclear network’s centripetal collapse. The authors support this with multiple independent lines of evidence: myosin II inhibitors do not block contraction, jasplakinolide (blocks depolymerization) prevents contraction, latrunculin (promotes net depolymerization) accelerates it, and formin dilution after NEBD shifts the balance toward disassembly. Second, although myosin-10 helps place chromosomes at the nuclear rim during prophase, during the post-NEBD contraction, chromosomes are largely coupled to the network by steric trapping—network pore sizes are far smaller than chromosome dimensions, making escape unlikely. Together these ideas expand our conception of force generation and object coupling in cellular systems.

Comparing actin mechanisms across development reveals marked adaptations. In starfish oocytes, chromosome congression relies on myosin II-driven contraction [11], whereas early mouse embryos utilize depolymerization-driven contraction. This difference may reflect adaptation to the rapid, synchronous cell cycles of embryonic development, where myosin-independent mechanisms could be more readily regulated. Additionally, while mammalian oocytes employ actin for chromosome compression [13,15], the persistence of nuclear actin from interphase into mitosis represents a distinct mechanism.

This study reframes the mitotic landscape in large, acentrosomal embryonic cells. Instead of a microtubule-only narrative, the authors show that actin provides a backup and even a frontline mechanism for chromosomal organization and spindle regulation. That explains how early mammalian embryos achieve surprisingly robust segregation despite weak centrosomal machinery and very large chromosomes. The findings challenge the conventional view that polar ejection forces alone drive chromosome congression [14,15], particularly in systems with large chromosomes and low microtubule density. The findings also bridge meiotic and mitotic literature on actin’s roles but with novel twists: nuclear interphase filaments persist into mitotic entry and are harnessed by disassembly-driven contraction, and Ran-dependent Arp2/3 activation sculpts a peri-spindle brake. These mechanistic insights could have downstream relevance for understanding the sources of embryonic aneuploidy and for improving assisted reproductive technologies by highlighting cytoskeletal vulnerabilities in early development. The discovery also raises broader cell-biological questions about when and where depolymerization-driven mechanics are used across life and how steric trapping might be a general strategy for moving large cargoes in crowded cellular spaces.

Hernandez et al. provide compelling evidence in mouse embryos and report similar nuclear actin in donated human embryos, suggesting conservation; however, several questions remain. First, does the peri-spindle actin mechanism operate in one-cell mouse embryos, where parental genome integration and spindle formation present unique challenges? Second, what are the regulatory differences between acentrosomal embryos and those with functional centrosomes? Third, in human embryos, do sperm-derived centrioles compete or cooperate with actin-based mechanisms, and does this vary across species? The species-specific dependency on actin mechanisms warrants further investigation. Clinically, abnormal actin dynamics could contribute to aneuploidy in assisted reproduction. Developing noninvasive markers of actin integrity might improve embryo selection.

The molecular regulation of formin sequestration/dilution and the upstream cues that spatially control anillin and Arp2/3 in the embryo deserve deeper biochemical work. Finally, how actin-based mechanisms integrate with nascent microtubule nucleation and kinetochore–microtubule error correction remains a fertile area for follow-up. Future research should focus on identifying the upstream regulators of formin and anillin dynamics, exploring potential cross talk between nuclear and peri-spindle networks, and validating these mechanisms in human embryogenesis.

By combining advanced live imaging with precise perturbations and quantitative analysis, Hernandez et al. reveal that actin is not merely a supporting actor in early embryonic mitosis; it is a central organizer. Two distinct actin assemblies compensate for weak centrosomal function by first corralling chromosomes through a depolymerization-driven nuclear network and then tamping spindle growth with an Arp2/3-dependent peri-spindle shell. This work overturns a microtubule-centric orthodoxy for a crucial developmental window and opens fresh avenues for thinking about cell division mechanics, reproductive biology, and the cellular design principles that preserve genome integrity when canonical machinery is absent.
